# Supplementary material for: iMUT-seq: high-resolution DSB-induced mutation profiling reveals prevalent homologous-recombination dependent mutagenesis
Source: Nat Commun. 2023 Dec 18;14:8419. doi: 10.1038/s41467-023-44167-1 (PMC10728174; doi:10.1038/s41467-023-44167-1)
Supplement: Supplementary file 1 — Supplementary Information [file 41467_2023_44167_MOESM1_ESM.pdf]

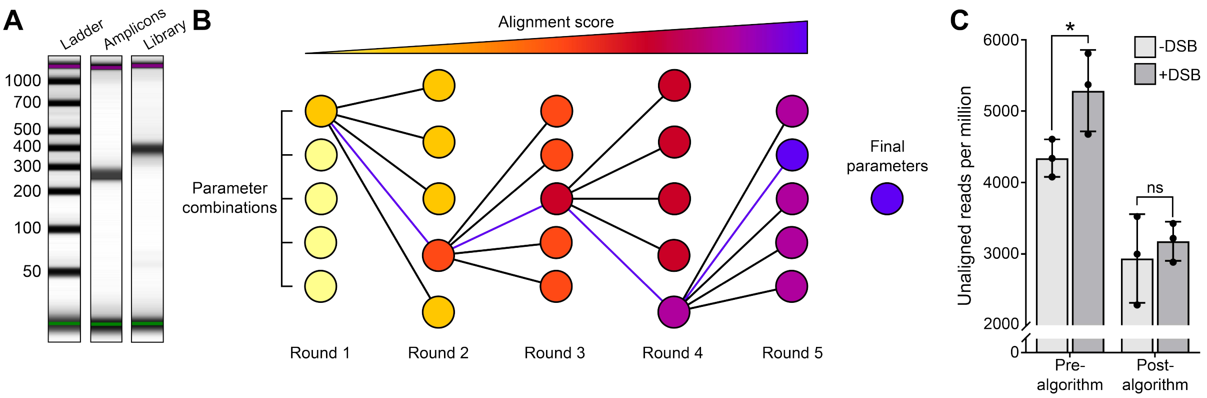

Supplementary Fig. 1: **iMUT-seq technique and analysis design. (A)** Agilent Tapestation gel image of iMUT-seq genomic amplicons and iMUT-seq final library, the amplicons are an average of ~270bp whereas the final library is ~400bp due to the addition of the Illumina adapters. **(B)** Diagrammatic depiction of the machine learning approach used to optimise the iMUT-seq alignments. Several alignment parameters that alter how mismatches, deletions and insertions are handled by the aligner can be optimised to improve alignment efficiency. The machine learning tool started with several different combinations of the parameters to be optimised, and used a genetic algorithm to procedurally improve the alignment over multiple rounds of testing. After each round, the parameter combination with the highest alignment score was carried forward and a new set of combinations was generated based off of this high scoring combination. Therefore, after each round the alignment got increasingly effective, until the optimal parameter settings were achieved. **(C)** Comparison of alignment efficiency pre- and post-optimisation of the alignment using the machine learning approach from (B), each point represents an independent biological replicate of iMUT-seq and error bars are S.D., n=3 independent biological replicates. Pre-algorithm, the number of unaligned reads with DSB induction was significantly higher than without DSB induction, indicating that DSB-induced mutations are reducing alignment efficiency. Whereas post-algorithm, there was no significant difference in the number of unaligned reads between with and without DSB induction. Source data and statistics are provided with this paper.

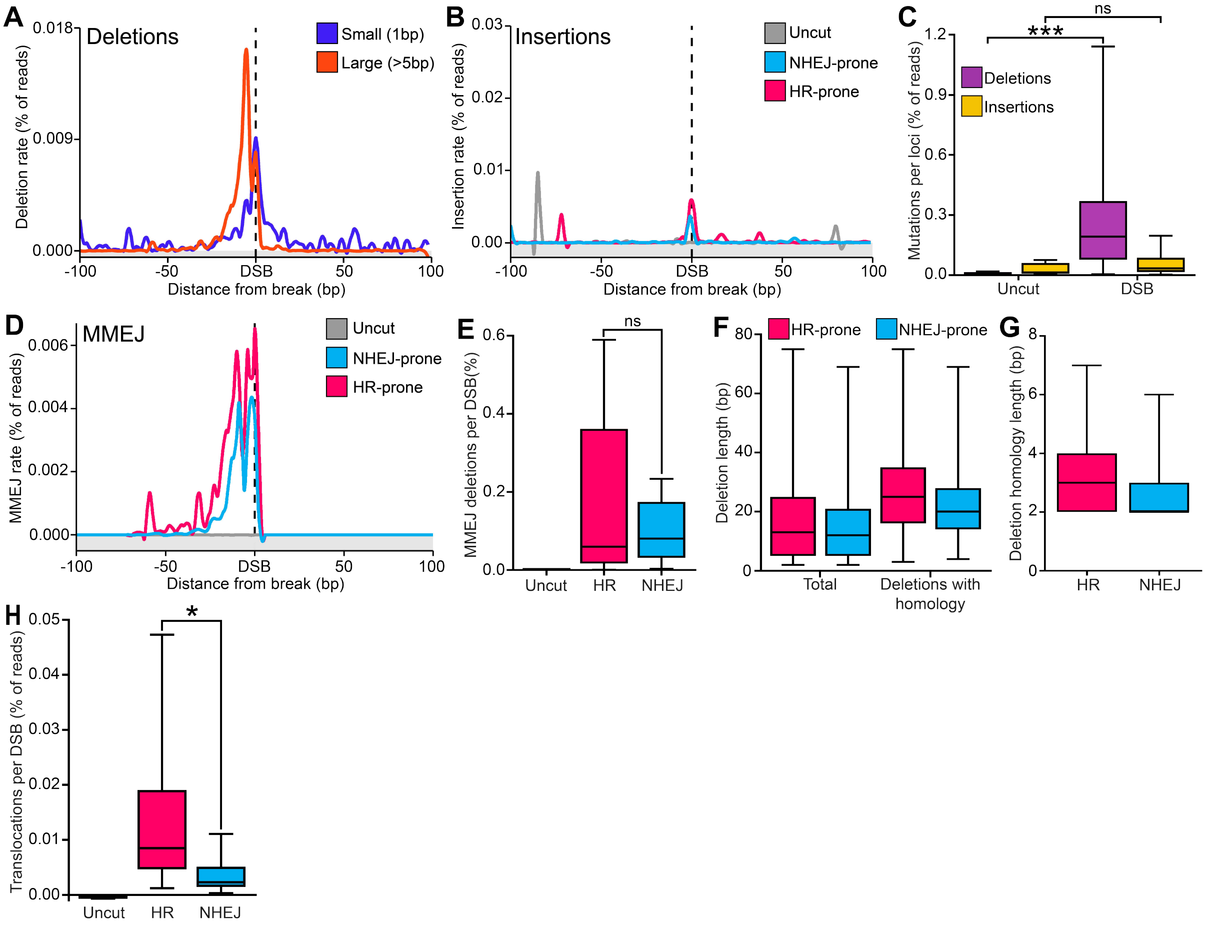

Supplementary Fig. 2: **iMUT-seq profiling of mutations at HR and NHEJ-prone DSBs.** **(A)** Metagene line plot of small and large deletion rate, as a percentage of readcount, 100bp either side of all AsiSI induced DSBs quantified by iMUT-seq **(B)** Same as (A) but for insertions at DSBs prone to repair by either NHEJ or HR, or at uncut control loci. **(C)** Boxplot of total deletions or insertions per loci as a percentage of readcount at either uncut control or DSB loci quantified by iMUT-seq, statistics done using a unpaired Wilcoxon test, n=20 loci for DSB and 5 for Uncut sites determined from 6 independent biological replicates. **(D)** Same as (A) but for microhomology-mediated end-joining (MMEJ) rates, deletions are reported at the first nucleotide of the deletion resulting in the peak skewing to the left of the DSB. **(E)** Boxplot of MMEJ rates per loci at either uncut control, HR-prone or NHEJ-prone loci quantified by iMUT-seq, n=10 loci for HR and NHEJ sites determined from 6 independent biological replicates. **(F)** Boxplot of deletion lengths at HR-prone or NHEJ-prone DSB loci for either total deletions or specifically deletions that show microhomologies, n=7621, 5592,

1204, 858 observed deletions respectively determined across 6 independent biological replicates. **(G)** Boxplot of microhomology lengths at DSB loci prone to either NHEJ or HR repair, n=3452, 2650 observed homologies respectively determined across 6 independent biological replicates. **(H)** Same as (E) but for translocation rates, statistics done via unpaired Wilcoxon test, * $p<0.05$. individual replicate values, error bar is standard deviation, statistics were done using paired t-test, ** $p<0.01$. **(I)** Same as (H) but for the DR-GFP HR repair reporter assay, *** $p<0.001$, n=10 loci for HR and NHEJ sites determined from 6 independent biological replicates. Source data and statistics are provided with this paper.

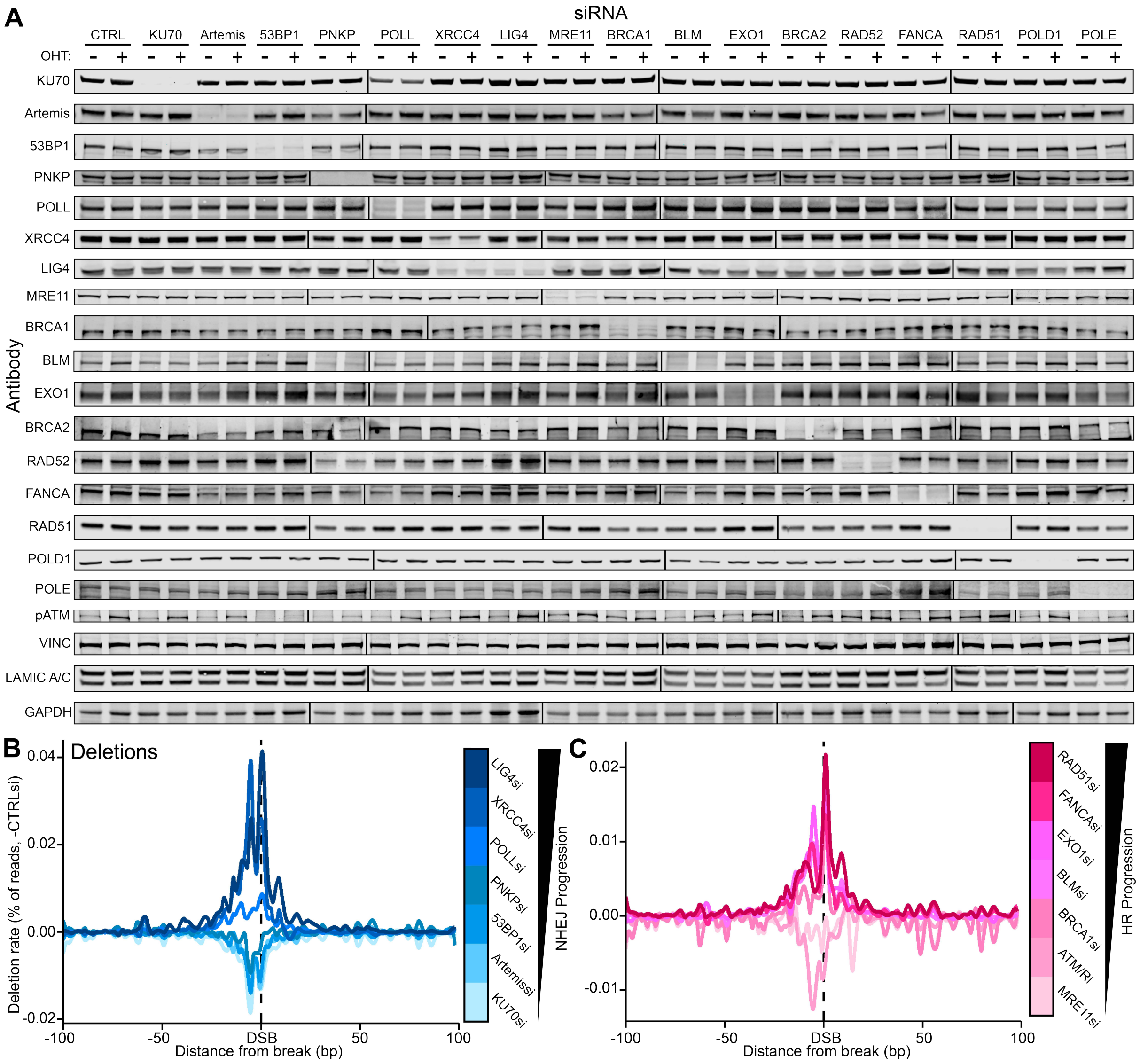

Supplementary Fig. 3: **Interrupting DSB repair causes increasing mutation rates the later the interruption occurs in the pathway. (A)** Western blot validation of the knockdown of 19 different DSB repair factors from both NHEJ and HR repair, in AID-DIvA cells with and without OHT treatment for 4 hours to induce DSBs. Due to the large number of samples, each blot had to be spread across 3-5 gels, and to maintain comparability all blots for the same target were ran, probed and scanned together, then treated identically during image processing, and lines were added to define the separate gels. Phosphorylated ATM was probed as a measure of DSB induction and one of three loading controls; GAPDH, Lamin-A/C or Vinculin was used during each western blot. **(B)** Metagene line plots of deletion rate delta to -DSB then delta to control siRNA, 100bp either side of AsiSI induced DSBs upon

knockdown of several different NHEJ repair factors, quantified by iMUT-seq. Legend (right) shows the colours for each siRNA target, as well as a bar depicting the position of the factors in the progression of NHEJ repair. **(C)** Same as (B) but with the knockdown of several HR repair factors. Source data are provided with this paper.

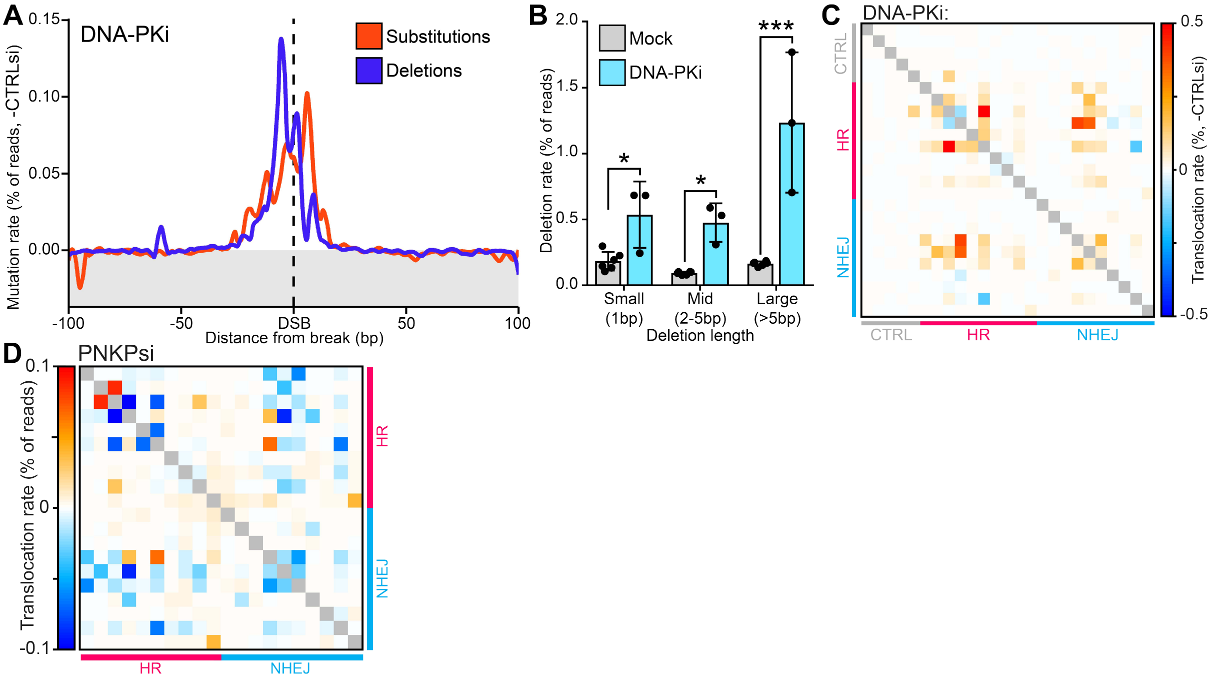

Supplementary Fig. 4: **DNA-PK inhibition causes a substantial increase in large deletions at DSBs. (A)** Metagene line plot of base substitution and deletion rates as a percentage of readcount delta to -DSB and then delta of DNA-PKi (10μM NU7441) – mock treatment, 100bp either side of AsiSI induced DSBs, quantified by iMUT-seq. **(B)** Bar plot of the rate of different deletion lengths per DSB loci as a percentage of readcount, quantified by iMUT-seq, with or without DNA-PKi treatment, points represent each biological replicate and error bars are S.D., statistics done using a paired t-test, * $p<0.05$, *** $p<0.001$, n=6 independent biological replicates for Mock and 3 for DNA-PKi treatment. **(C)** Heatmap of translocation rates between different DSBs quantified by iMUT-seq with DNA-PKi treatment, each row and column represent a different iMUT-seq amplicon, each cell representing the translocation rate calculated between the row/column amplicons as a percentage of readcount delta to -DSB and then delta to mock treatment. **(D)** Same as (C) but for PNKPsi treatment at HR and NHEJ prone loci. Source data and statistics are provided with this paper.

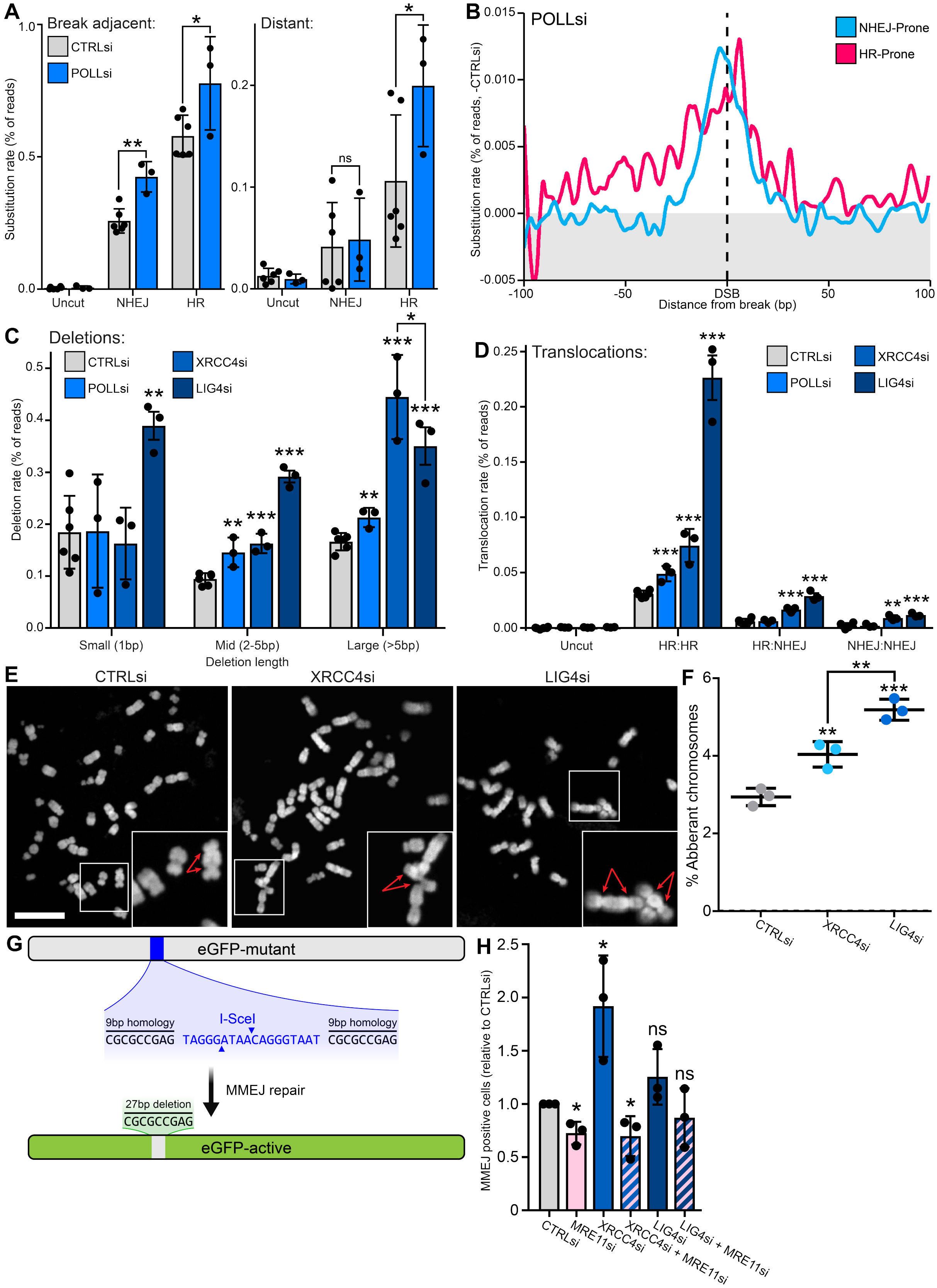

Supplementary Fig. 5: Disruption of late NHEJ processes promotes large deletions and global translocations. (A) Bar plots of base substitution rates at either uncut loci or DSB

loci prone to either NHEJ or HR repair, comparing CTRLsi to POLLsi, left shows substitutions adjacent to the break point and right shows substitutions distant from the break site, points represent each biological replicate and error bars are S.D., statistics done using a paired t-test, * $p<0.05$, ** $p<0.01$, n=6 independent biological replicates for CTRLsi and 3 for POLLsi. **(B)** Metagene line plot of base substitution rate at DSB loci prone to either NHEJ or HR repair with POLL depletion. **(C)** Bar plot of the rate of different deletion lengths per DSB loci as a percentage of readcount, quantified by iMUT-seq, with siRNA depletion of either POLL, XRCC4 or LIG4, points represent each biological replicate and error bars are S.D., statistics done relative to CTRLsi or between XRCC4si and LIG4si using a paired t-test, ** $p<0.01$, *** $p<0.001$, n=6 independent biological replicates for CTRLsi and 3 for all other conditions. **(D)** Average translocation rate between different DSBs quantified by iMUT-seq, split by events between sites repaired by either HR or NHEJ or uncut control loci, treated with either control, POLL, XRCC4 or LIG4 siRNA, points represent each biological replicate and error bars are S.D., all statistics done relative to CTRLsi using paired t-tests, ** $p<0.01$, *** $p<0.001$, n=6 independent biological replicates for CTRLsi and 3 for all other conditions. **(E)** Representative images of metaphase spreads with 1hr 20μM etoposide treatment and with either control, XRCC4 or LIG4 siRNA, scalebar is 5μm. **(F)** Quantification of chromosomal aberrations in the metaphase spreads from (E) as a percentage of total chromosomes, points represent each biological replicate and error bars are S.D., statistics done relative to control siRNA using a paired t-test, ** $p<0.01$, *** $p<0.001$, n=3 independent biological replicates. **(G)** Schematic diagram of the GFP based MMEJ reporter assay. **(H)** Results of the MMEJ reporter assay in (G) with either MRE11, XRCC4 or LIG4 siRNA mediated depletion or combinations of these, all data is normalised to control siRNA, error bars are S.D., statistics done relative to control siRNA using a paired t-test, * $p<0.05$, n=3 independent biological replicates. Source data and statistics are provided with this paper.

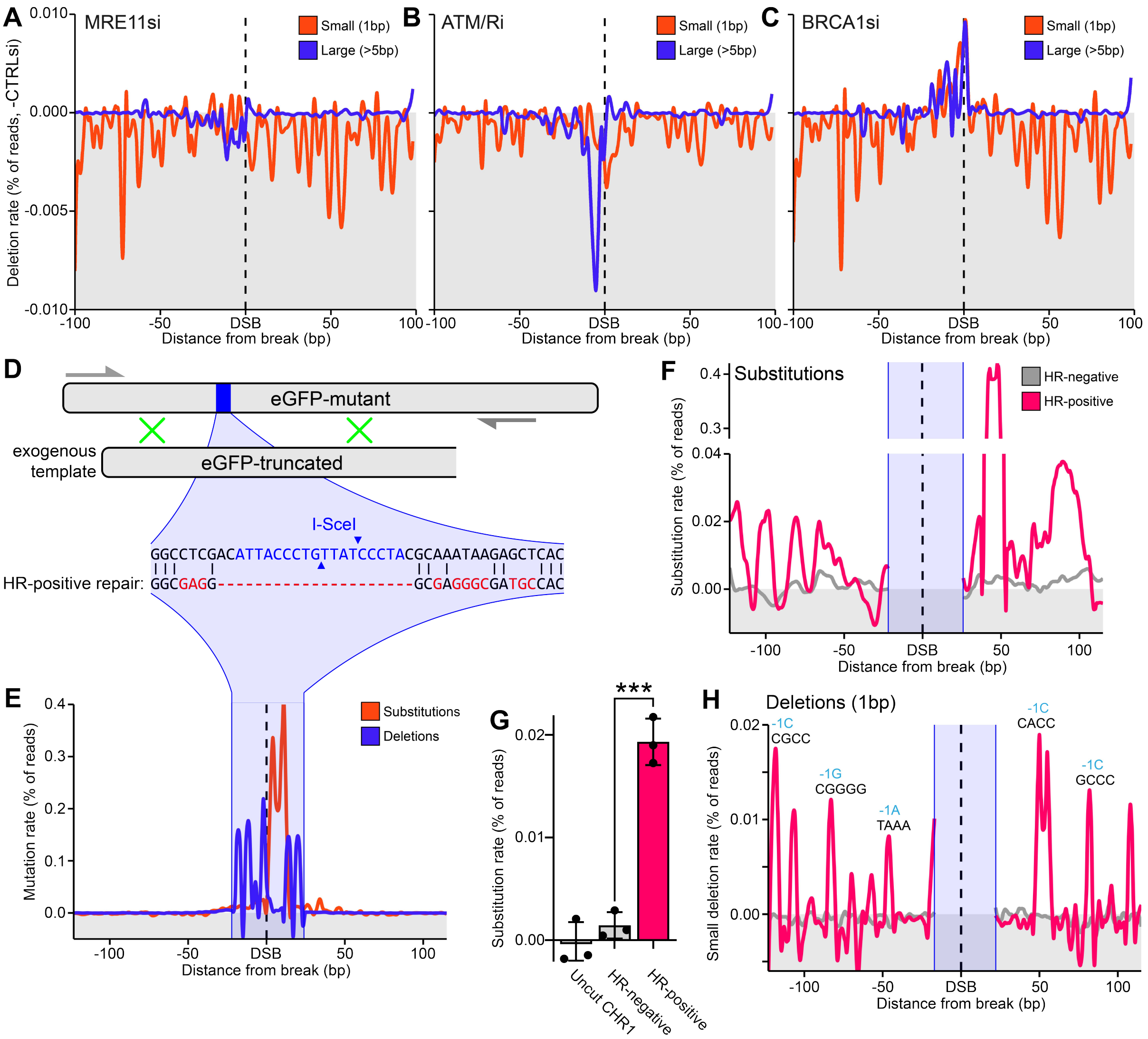

Supplementary Fig. 6: Early HR processes promote deletions at DSBs. (A) Metagene line plot of small (1bp) and large (>5bp) deletion rates as a percentage of readcount delta to -DSB and then delta of MRE11 siRNA – control siRNA, 100bp either side of AsiSI induced DSBs, quantified by iMUT-seq. **(B)** Same as (A) but for ATM/ATR inhibition. **(C)** same as (A) but for BRCA1 depletion. **(D)** Schematic diagram of the traffic light reporter (TLR) assay for homologous recombination. **(E)** Metagene line plot of base substitutions and deletions around the DSB in the TLR-reporter assay shown in (D) with the region altered via homologous recombination highlighted. **(F)** Metagene line plot of base substitutions around the DSB in the TLR-reporter assay with the region directly altered by homologous

recombination excluded, where reads were categorised into either HR-positive or HR-negative. **(G)** Average per nucleotide substitution rate at either uncut control loci or the cut TLR-reporter assay locus in either HR-positive or HR-negative reads, error bars are S.D., statistics done using a paired t-test, *** $p<0.001$, $n=3$ independent biological replicates. **(H)** Same as (F) but for deletions and with the nucleotide context of the peaks. Source data and statistics are provided with this paper.

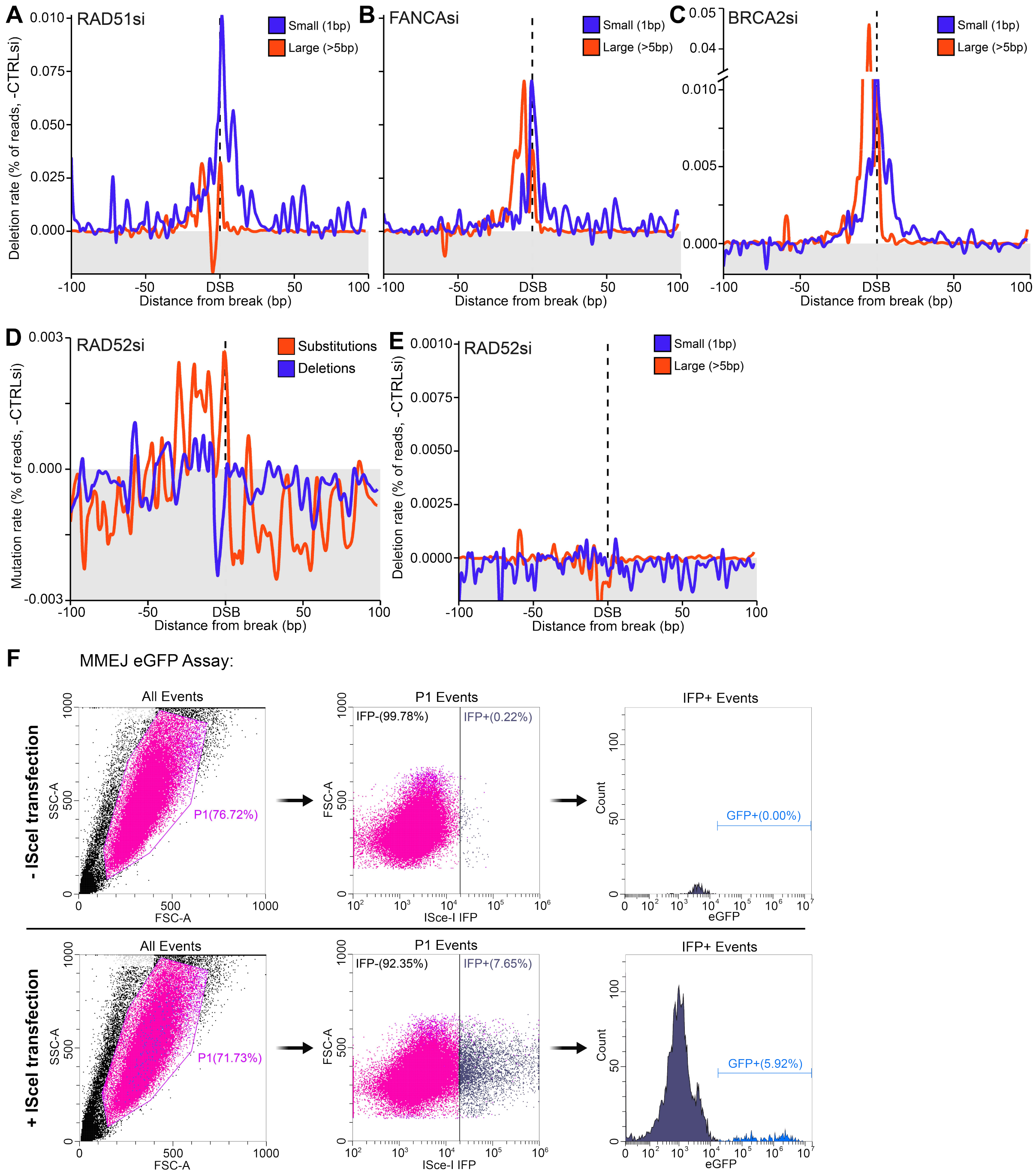

Supplementary Fig. 7: The effects of depletion of BRCA2, FANCA, RAD52 and RAD51 on DSB-induced deletion lengths and base substitution signatures. (A) Metagene line plot of small (1bp) and large (>5bp) deletion rates as a percentage of readcount delta to -DSB and then delta of RAD51 siRNA – control siRNA, 100bp either side of AsiSI induced DSBs, quantified by iMUT-seq. **(B)** Same as (A) but for FANCA siRNA. **(C)** Same as (A) but for BRCA2 siRNA. **(D)** Metagene line plot with RAD52 depletion of base substitution and deletion rates delta to -DSB and then delta to control siRNA. **(E)** Same as (A) but for RAD52

siRNA. Source data are provided with this paper. Representative images demonstrating the gating procedure used for MMEJ-GFP assay, cells were first selected using an FSC/SSC gate before selecting ISce-I IFP expressing cells based on IFP signal and from this population of cells the percentage of GFP-positive cells was calculated using an eGFP gate. Both the IFP and eGFP gates were calibrated based on a sample that was not transfected with ISce-I IFP.
