# Supplementary material for: iMUT-seq: high-resolution DSB-induced mutation profiling reveals prevalent homologous-recombination dependent mutagenesis
Source: Nat Commun. 2023 Dec 18;14:8419. doi: 10.1038/s41467-023-44167-1 (PMC10728174; doi:10.1038/s41467-023-44167-1)
Supplement: Supplementary file 3 — Description of Additional Supplementary Files Document [file 41467_2023_44167_MOESM3_ESM.pdf]

Description of Additional Supplementary Files

Supplementary Datasets

Supplementary Data 1: Genomic primer pairs for iMUT-seq with metadata.
Supplementary Data 2: siRNA targets, sequences and sources.
Supplementary Data 3: Antibodies, sources and catalog numbers
