# Supplementary material for: iMUT-seq: high-resolution DSB-induced mutation profiling reveals prevalent homologous-recombination dependent mutagenesis
Source: Nat Commun. 2023 Dec 18;14:8419. doi: 10.1038/s41467-023-44167-1 (PMC10728174; doi:10.1038/s41467-023-44167-1)
Supplement: Supplementary file 7 — Reporting Summary [file 41467_2023_44167_MOESM7_ESM.pdf]

nature portfolio

Corresponding author(s): Martin Bushell
Aldo S Bader

Last updated by author(s): Aug 10, 2023

Reporting Summary

Nature Portfolio wishes to improve the reproducibility of the work that we publish. This form provides structure for consistency and transparency in reporting. For further information on Nature Portfolio policies, see our Editorial Policies and the Editorial Policy Checklist.

Statistics

For all statistical analyses, confirm that the following items are present in the figure legend, table legend, main text, or Methods section.

n/a	Confirmed	
☐	☒	The exact sample size (*n*) for each experimental group/condition, given as a discrete number and unit of measurement
☐	☒	A statement on whether measurements were taken from distinct samples or whether the same sample was measured repeatedly
☐	☒	The statistical test(s) used AND whether they are one- or two-sided<br>*Only common tests should be described solely by name; describe more complex techniques in the Methods section.*
☐	☒	A description of all covariates tested
☐	☒	A description of any assumptions or corrections, such as tests of normality and adjustment for multiple comparisons
☐	☒	A full description of the statistical parameters including central tendency (e.g. means) or other basic estimates (e.g. regression coefficient) AND variation (e.g. standard deviation) or associated estimates of uncertainty (e.g. confidence intervals)
☐	☒	For null hypothesis testing, the test statistic (e.g. *F*, *t*, *r*) with confidence intervals, effect sizes, degrees of freedom and *P* value noted<br>*Give P values as exact values whenever suitable.*
☒	☐	For Bayesian analysis, information on the choice of priors and Markov chain Monte Carlo settings
☒	☐	For hierarchical and complex designs, identification of the appropriate level for tests and full reporting of outcomes
☒	☐	Estimates of effect sizes (e.g. Cohen's *d*, Pearson's *r*), indicating how they were calculated

Our web collection on statistics for biologists contains articles on many of the points above.

Software and code

Policy information about availability of computer code

Data collection	The only software based data collection was via the machine learning script that was used to optimise the Bowtie2 alignment. The software used for this is available in the GitHub repository for this manuscript https://github.com/aldob/iMUT-seq
Data analysis	All analytical code is publicly available on GitHub (https://github.com/aldob/iMUT-seq), which includes the raw data processing pipeline and it's parameters as well as the code used to generate plots in R. Software versions: samtools 1.6, bowtie2 2.2.5, fastp 0.22.0, imagej 1.54, mProfile 1.4

For manuscripts utilizing custom algorithms or software that are central to the research but not yet described in published literature, software must be made available to editors and reviewers. We strongly encourage code deposition in a community repository (e.g. GitHub). See the Nature Portfolio guidelines for submitting code & software for further information.

Data

Policy information about availability of data

All manuscripts must include a data availability statement. This statement should provide the following information, where applicable:

- Accession codes, unique identifiers, or web links for publicly available datasets
- A description of any restrictions on data availability
- For clinical datasets or third party data, please ensure that the statement adheres to our policy

All iMUT-seq raw data has been deposited at ArrayExpress under the accession E-MTAB-11259 and is publicly accessible.

Research involving human participants, their data, or biological material

Policy information about studies with human participants or human data. See also policy information about sex, gender (identity/presentation), and sexual orientation and race, ethnicity and racism.

Reporting on sex and gender	N/A
Reporting on race, ethnicity, or other socially relevant groupings	N/A
Population characteristics	N/A
Recruitment	N/A
Ethics oversight	N/A

Note that full information on the approval of the study protocol must also be provided in the manuscript.

Field-specific reporting

Please select the one below that is the best fit for your research. If you are not sure, read the appropriate sections before making your selection.

☒ Life sciences ☐ Behavioural & social sciences ☐ Ecological, evolutionary & environmental sciences

For a reference copy of the document with all sections, see nature.com/documents/nr-reporting-summary-flat.pdf

Life sciences study design

All studies must disclose on these points even when the disclosure is negative.

Sample size	No statistical method was used to determine sample sizes, all samples sizes follow normal convention for comparable studies; 3 biologically independent replicates to provide statistically relevant results.
Data exclusions	No data exclusions have been performed.
Replication	All experiments are done in at least biological triplicate. These replicates were done completely separately on separate days and individual replicate values are often represented in the figures.
Randomization	No randomisation method was used. Conditions were split across three batches with no consistent attribute within the groups. Group allocation was via a combination of experimental practicality and changing target choices across the course of the project.
Blinding	For metaphase spread and IF experiments, samples were blinded for data collection and analysis by having the labels changed after sample preparation by a researcher not involved in the experiment. For other experiments blinding was not used. Whereas sample names were not blinded for sequencing experiments, the analysis is conducted in an automated manner, and therefore has no potential for bias.

Reporting for specific materials, systems and methods

We require information from authors about some types of materials, experimental systems and methods used in many studies. Here, indicate whether each material, system or method listed is relevant to your study. If you are not sure if a list item applies to your research, read the appropriate section before selecting a response.

Materials & experimental systems

n/a	Involved in the study	
☐	☒	Antibodies
☐	☒	Eukaryotic cell lines
☒	☐	Palaeontology and archaeology
☒	☐	Animals and other organisms
☒	☐	Clinical data
☒	☐	Dual use research of concern
☒	☐	Plants

Methods

n/a	Involved in the study	
☒	☐	ChIP-seq
☐	☒	Flow cytometry
☒	☐	MRI-based neuroimaging

Antibodies

Antibodies used	Mouse monoclonal anti-KU70, SCBT, sc-5309, 1/2000<br>Rabbit polyclonal anti-Artemis, Novus Biologicals, NBP2-15477, 1/500<br>Rabbit polyclonal anti-53BP1, Novus Biologicals, NB100-305, 1/1000<br>Mouse monoclonal anti-PNKP, SCBT, sc-166153, 1/500<br>Mouse monoclonal anti-POLL, SCBT, sc-373844, 1/1000<br>Mouse monoclonal anti-XRCC4, SCBT, sc-271087, 1/500<br>Rabbit polyclonal anti-LIG4, Proteintech, 12695-1-AP, 1/1000<br>Mouse monoclonal anti-MRE11, SCBT, sc-135992, 1/500<br>Mouse monoclonal anti-BRCA1, SCBT, sc-6954, 1/1000<br>Rabbit polyclonal anti-BLM, Abcam, Ab2179, 1/500<br>Rabbit polyclonal anti-EXO1, Proteintech, 16253-1-AP, 1/500<br>Mouse monoclonal anti-BRCA2, Sigma-Aldrich, OP95, 1/500<br>Rabbit polyclonal anti-FANCA, Bethyl, A301-980A, 1/5<br>Rabbit polyclonal anti-RAD52, Proteintech, 28045-1-AP, 1/1000<br>Rabbit polyclonal anti-RAD51, Abcam, ab176458, 1/2000<br>Mouse monoclonal anti-RAD51, Novus Biologicals, NB100-148, 1/250<br>Rabbit monoclonal anti-RPA70, Abcam, ab79398, 1/500<br>Mouse monoclonal anti-POLD1, SCBT, sc-374025, 1/1000<br>Mouse monoclonal anti-POLE, Novus Biologicals, NB100-115, 1/500<br>Rabbit polyclonal anti-Lamin A/C, CST, 2032T, 1/2000<br>Rabbit monoclonal anti-Vinculin, Abcam, ab129002, 1/5000<br>Rabbit monoclonal anti-GAPDH, CST, 5174S, 1/5000<br>Rabbit monoclonal anti-phospho ATM (S1981), Abcam, ab81292, 1/500<br>Goat polyclonal anti-mouse IgG Alexa Fluor 594, Abcam, ab150116, 1/1000<br>Goat polyclonal anti-rabbit IgG Alexa Fluor 594, Abcam, ab150080, 1/1000<br>Goat polyclonal anti-mouse IgG Alexa Fluor 488, Abcam, ab150113, 1/1000<br>Goat polyclonal anti- rabbit IgG Alexa Fluor 488, Abcam, ab150077, 1/1000<br>IRDye 800CW Goat anti-Mouse IgG, Li-Cor, 926-32210, 1/10000<br>IRDye 680CW Goat anti-Rabbit IgG, Li-Cor, 926-68071, 1/10000
Validation	Most antibodies were validated via siRNA depletion or appropriate treatments within the manuscript, Supplemental Fig. 3a, additional validations can be found on the manufacturer websites.

Eukaryotic cell lines

Policy information about cell lines and Sex and Gender in Research

Cell line source(s)	U2OS, HCT-166 and RPE-1 cells were all obtained from ATCC, AID-DIvA cells were received from the Gaëlle Legube Lab.
Authentication	Cell lines have not been authenticated.
Mycoplasma contamination	All cell lines were tested for mycoplasma contamination each time they entered cell culture from storage and were always found to be negative.
Commonly misidentified lines<br>(See ICLAC register)	HCT-116 cells are a commonly misidentified cell line, however in our hands they showed an accurate karyotype and behaved as expected. HCT-116 cells were used for some metaphase spread experiments as they have a consistent karyotype while also being a transformed cell line with the potential for aneuploidies allowing for a large therapeutic window for our experiments. The cell line also performs well in sample preparation for metaphase spreads. All significant findings with HCT116 cells were also validated with RPE-1 cells.

Flow Cytometry

Plots

Confirm that:

☒ The axis labels state the marker and fluorochrome used (e.g. CD4-FITC).

☒ The axis scales are clearly visible. Include numbers along axes only for bottom left plot of group (a 'group' is an analysis of identical markers).

☒ All plots are contour plots with outliers or pseudocolor plots.

☒ A numerical value for number of cells or percentage (with statistics) is provided.

Methodology

Sample preparation	500,000 U2OS MMEJ reporter cells were seeded onto 10cm plates and transfected with siRNA as described earlier. 48 hours after siRNA transfection, cells were transfected with a plasmid encoding the I-SceI endonuclease (Addgene #31482) using Lipofectamine 2000 (ThermoFisher, #11668019) according to the manufacturer instructions. Cells were incubated for a

further 72 hours before harvesting by trypsinisation. Cells were pelleted at 300g, washed once in PBS, re-pelleted and then analysed.

Instrument	Beckman Coulter CytoFLEX
Software	Beckman Coulter CytExperty 2.4.0.28
Cell population abundance	Cells were not sorted and therefore purity calculations of sorted fractions is not relevant.
Gating strategy	Single cells were initially gated for using FSC/SSC. Single cells were selected by IFP expression of the I-SceI encoding plasmid before quantification of the GFP-positive cells to prevent variations in transfection efficiency from impacting our results. To gate for IFP and GFP, un-transfected and transfected control negative samples were used, for GFP, an eGFP encoding plasmid was transfected as a positive control to one sample.

☒ Tick this box to confirm that a figure exemplifying the gating strategy is provided in the Supplementary Information.
